# Gut dysbiosis in difficult-to-treat rheumatoid arthritis: hypothesized microbial endotypes, persistent inflammation, and pharmacological treatment resistance

**DOI:** 10.3389/fphar.2026.1939732

**Published:** 2026-09-22

**Authors:** Zhibo Chai, Mengyi Wang, Jiannan Zheng, Jing Yu

**Affiliations:** 1 The First Clinical College, Liaoning University of Traditional Chinese Medicine, Shenyang, China; 2 Department of Rheumatology and Immunology, Affiliated Hospital of Liaoning University of Traditional Chinese Medicine, Shenyang, China

**Keywords:** difficult-to-treat rheumatoid arthritis, disease-modifying antirheumatic drugs, gut microbiota, microbial endotypes, persistent inflammation, pharmacomicrobiomics, treatment resistance

## Abstract

Difficult-to-treat rheumatoid arthritis (D2T RA) remains a major clinical problem despite treat-to-target care and an expanding range of disease-modifying antirheumatic drugs (DMARDs). D2T RA includes biologically distinct states, particularly persistent inflammatory refractory rheumatoid arthritis (PIRRA) and non-inflammatory refractory rheumatoid arthritis (NIRRA). Direct microbiome evidence in D2T RA is sparse, but studies in established RA, at-risk populations, and treatment-response cohorts suggest that intestinal ecological disruption may sustain inflammation through barrier failure, persistent microbial-product sensing, and impaired metabolite-mediated immune regulation. Gut microorganisms may also influence drug response, especially to methotrexate, while antirheumatic therapy can remodel the microbial ecosystem. We therefore synthesize the literature into three hypothesis-generating functional states, provisionally termed inflammation-amplifying, immune-tolerance-deficient, and poor-drug-response-associated microbial endotypes. These states are not validated patient classes and may overlap or evolve over time. We also propose a microbiota–treatment resistance feedback loop, explicitly as an inferential model rather than an established causal pathway. Microbiota-directed adjuncts are evaluated according to evidence maturity, mechanistic fit, and safety. Overall, the gut microbiota is best considered a potential upstream modifier linking mucosal immunity, persistent inflammation, and pharmacological response, with the most coherent mechanistic relevance to PIRRA. Prospective D2T RA cohorts with objective inflammatory phenotyping, longitudinal multi-omics, and detailed drug-exposure data are needed before these concepts can inform clinical stratification or treatment.

## 1 Introduction

Rheumatoid arthritis (RA) management is now centered on early DMARD initiation, treat-to-target monitoring, and timely treatment adjustment. Current EULAR recommendations and ACR guidance retain conventional synthetic DMARDs (csDMARDs), particularly methotrexate (MTX), as the usual therapeutic foundation, with biological and targeted synthetic DMARDs (bDMARDs and tsDMARDs) used when treatment targets are not achieved (Fraenkel et al., 2021; Smolen et al., 2026). Even with this broader pharmacological repertoire, a clinically important subgroup remains uncontrolled. The EULAR definition identifies D2T RA through three linked domains: failure of csDMARDs and at least two bDMARDs or tsDMARDs with different mechanisms of action, ongoing active or symptomatic disease, and management that remains problematic for the patient and/or rheumatologist (Nagy et al., 2021). Registry and meta-analytic estimates place this population at approximately 10%–15% in many treated cohorts, although prevalence varies with case mix and the implementation of the definition (Paudel et al., 2025; Xie et al., 2026).

D2T RA is an operational clinical construct rather than a biologically uniform subtype. Persistent objective inflammation, pain sensitization, fatigue, structural damage, comorbidity, psychological factors, adherence, and cumulative drug toxicity can all produce an apparently refractory course (Nagy and Buch, 2025). The distinction between PIRRA and NIRRA is therefore central to mechanistic interpretation. PIRRA is characterized by continuing synovitis or other objective inflammatory activity despite multiple therapies, whereas NIRRA is dominated by pain, fatigue, disability, or elevated composite scores that are disproportionate to demonstrable inflammation (Buch et al., 2021). Synovial pathophenotyping lends biological support to this distinction: immune-rich lympho-myeloid or diffuse-myeloid patterns are more frequent in PIRRA, while pauci-immune or fibroid patterns are more common in NIRRA (Giollo et al., 2026). These phenotypes overlap and may change over time, but they should not be pooled uncritically in microbiome studies.

Microbiome research offers a plausible way to examine upstream processes that are not captured by a single cytokine or cellular target. RA-associated dysbiosis is better understood as altered community function than as the uniform expansion or depletion of one organism. Experimental and human studies implicate barrier injury, microbial metabolites, mucosal immune imbalance, and translocation-related signals. *Faecalibacterium prausnitzii* can modulate short-chain fatty acids (SCFAs) and IL-17-associated responses in experimental arthritis (Moon et al., 2023). The only direct comparison currently available reported compositional and predicted functional differences between D2T RA and easy-to-treat RA (E2T RA), including relative enrichment of *Lachnospiraceae* and *Coprococcus* in E2T RA and a lower *Firmicutes*/*Bacteroidetes* ratio in D2T RA (Ruiz-Limón et al., 2025). These findings are exploratory and should not be treated as a diagnostic signature. Broader RA studies further show oral–gut ecological disturbance (Zhang et al., 2015), strain-dependent pathogenicity of *Prevotella copri* (Jiang et al., 2022; Nii et al., 2023), barrier-related effects of SCFA-associated networks, and associations between pretreatment microbial function and DMARD response (Chen et al., 2016; Danckert et al., 2024; Luo et al., 2023).

This review asks whether these observations can be organized into a pharmacologically relevant framework for D2T RA. Direct D2T RA data define the clinical problem; conventional RA and experimental studies provide mechanistic context; and treatment-response cohorts support pharmacomicrobiomic hypotheses (Danckert et al., 2024; Romero-Figueroa et al., 2023; Ruiz-Limón et al., 2025). The mechanistic model is intentionally centered on PIRRA, where objective inflammation provides a measurable biological endpoint. NIRRA is treated as an important boundary condition: microbiota-related gut–brain effects may modify pain or fatigue, but current evidence does not support a microbiome-based explanation for NIRRA. Throughout, proposed “endotypes” are used as shorthand for hypothesized functional states, not data-derived biological classes.

### 1.1 Literature search and evidence synthesis

This article is a narrative, hypothesis-generating review rather than a systematic review. To improve methodological transparency, a targeted literature update was performed in PubMed/MEDLINE through 31 August 2026. Search concepts combined “rheumatoid arthritis” with “difficult-to-treat,” “refractory,” or “PIRRA,” and with “gut microbiota,” “microbiome,” “intestinal barrier,” “lipopolysaccharide,” “short-chain fatty acid,” “Th17,” “Treg,” “methotrexate,” “DMARD,” “TNF inhibitor,” “JAK inhibitor,” “probiotic,” “postbiotic,” or “fecal microbiota transplantation”; reference lists of key reviews and primary studies were also screened. Human D2T RA studies were prioritized, followed by longitudinal or treatment-response studies in conventional RA; animal and *in vitro* studies were included only to support mechanistic plausibility where direct human evidence was unavailable. We distinguished measured microbial composition, inferred or measured microbial function, and measured microbial metabolites, and treated oral-microbiome studies as indirect supporting evidence. No formal risk-of-bias tool or quantitative evidence grading was applied because the review was not designed as a systematic review or meta-analysis. Instead, evidence was interpreted according to study design, sample size, temporal information, directness to D2T RA, control of medication and host confounding, and whether microbial function was measured directly or inferred from 16S data.

## 2 Difficult-to-treat rheumatoid arthritis: definition, clinical phenotypes, and mechanistic uncertainty

### 2.1 The EULAR definition and its boundaries of application

EULAR defines D2T RA as an operational clinical construct rather than an independent disease subtype with a shared biological basis. Classification requires a reasonably guideline-concordant treatment pathway: an inadequate response to at least two bDMARDs or tsDMARDs with different mechanisms of action after csDMARD failure, unless contraindications prevent their use; persistent active or symptomatic disease, including moderate-to-high disease activity, signs or symptoms suggestive of active disease, inability to taper glucocorticoids, rapid radiographic progression, or a substantial RA-related reduction in quality of life; and a perception by the rheumatologist and/or patient that management remains problematic (Nagy et al., 2021). The strength of this definition is that it integrates treatment history, residual disease burden, and practical management difficulty rather than merely counting failed therapies. Subsequent EULAR points to consider also emphasize concurrent assessment of inflammatory activity, adherence, pain, fatigue, functional impairment, and comorbidities (Nagy et al., 2022). The definition is therefore well suited as a common entry point for clinical recognition and research enrollment, but it should not be equated with a single biological entity of treatment resistance. Mechanistic interpretation still requires further stratification according to inflammatory status, the source of symptoms, and overall clinical complexity (Hofman et al., 2025; Nagy and Buch, 2025).

### 2.2 Prevalence and disease burden of D2T RA

The epidemiological significance of D2T RA lies less in a single fixed prevalence estimate than in the repeated identification of this high-burden population across real-world treatment settings. In the BRASS registry, the prevalence of D2T RA was 14.4% in the overall RA population and 22.3% among those previously treated with bDMARDs or tsDMARDs (Paudel et al., 2025). The Korean KOBIO registry identified D2T RA in 11.7% of patients receiving bDMARDs or tsDMARDs (Jung et al., 2023), whereas an early RA cohort found that 35 of 631 patients (5.87%) progressed to D2T RA (Leon et al., 2023). A longitudinal analysis further showed that patients meeting the definition at a single time point did not fully overlap with those who remained in a D2T state for at least 6 months (Cincinelli et al., 2024). These differences primarily reflect variation in disease stage, cohort composition, exposure to targeted therapies, and implementation of the definition; they should not be interpreted as directly comparable fixed prevalence estimates. Importantly, D2T RA is more than sequential treatment failure. In the KOBIO study, patients maintained higher disease activity despite continued bDMARD or tsDMARD treatment and more frequently discontinued targeted therapy because of insufficient efficacy (Jung et al., 2023). Other studies indicate that adverse drug events, comorbidities, fibromyalgia, and limited coping capacity collectively increase disease burden (Roodenrijs et al., 2021a). D2T RA is also associated with greater healthcare utilization and annual costs, with functional disability being an important determinant of expenditure (Roodenrijs et al., 2021b). Long-term follow-up has linked a higher comorbidity burden and greater glucocorticoid exposure to increased mortality (Takanashi et al., 2025). D2T RA should therefore be regarded as a complex state in which inflammatory activity, functional impairment, glucocorticoid exposure, healthcare costs, and reduced quality of life converge.

### 2.3 PIRRA and NIRRA: a critical stratification for mechanistic research

Within the broad D2T RA construct, the first mechanistic question is whether refractoriness is driven predominantly by persistent inflammation or by non-inflammatory symptom burden. The PIRRA/NIRRA framework proposed by Buch and colleagues addresses this distinction: PIRRA denotes objective inflammation after multiple targeted therapies, whereas NIRRA describes persistent disease activity scores or symptoms that are not adequately explained by concurrent inflammation (Buch et al., 2021). PIRRA should therefore not be defined solely by the number of treatment failures or by composite disease activity scores, but by objective inflammatory evidence. Ultrasound studies have distinguished PIRRA from NIRRA according to the presence of synovitis and identified differences in swollen-joint counts, C-reactive protein (CRP), and the prevalence of fibromyalgia (David et al., 2024). Imaging follow-up further associated swollen-joint counts, CRP, and the PIRRA phenotype with rapid radiographic progression (Abacar et al., 2026). Synovial biopsy findings support this stratification: PIRRA more often features active synovitis, a higher inflammatory burden, and greater immune-cell infiltration, whereas NIRRA more commonly displays low-inflammatory, pauci-immune, or fibroid patterns (Giollo et al., 2026). Persistent symptoms in NIRRA are more likely to reflect a combination of fibromyalgia, comorbidities, limited coping capacity, and altered pain processing (Roodenrijs et al., 2021a). In RA, pain sensitization is associated with higher Clinical Disease Activity Index scores, tender-joint counts, and patient global assessments (Lee et al., 2018), and clinical and ultrasonographic inflammatory measures do not fully account for pain burden, supporting central sensitization as an additional mechanism (Georgopoulos et al., 2024). Against this background, the gut microbiota is most plausibly relevant to persistent inflammation in PIRRA. Barrier disruption, mucosal immune activation, Th17/Treg imbalance, altered metabolites, and translocation of microbial products can be mechanistically aligned with persistent synovitis, elevated inflammatory markers, and structural progression (Romero-Figueroa et al., 2023). The microbiota should not be positioned as a primary cause of NIRRA; nevertheless, pain and fatigue in RA are linked to central sensitization (Georgopoulos et al., 2026), and the gut-brain axis may modulate chronic pain and symptom perception (Goudman et al., 2024). Microbial factors could therefore influence a subset of non-inflammatory symptoms. PIRRA and NIRRA are not mutually exclusive, and phenotypes may change over time or with treatment; this dynamic overlap should inform all subsequent mechanistic interpretations.

### 2.4 Why microbiome and pharmacomicrobiomic perspectives are needed

Failure of therapies directed at multiple immune pathways raises the possibility that a subset of D2T RA is sustained by upstream, distributed inputs rather than by one dominant downstream target (Nagy et al., 2021). The intestinal microbiota is relevant because it can influence mucosal immunity, epithelial integrity, microbial metabolite availability, and drug handling at the same time. Direct D2T RA data remain sparse, but the available comparison with E2T RA identified differences in community composition and predicted metabolism (Ruiz-Limón et al., 2025). Independent human RA cohorts have also reported reduced fecal butyrate and propionate and depletion of SCFA-producing organisms (Blank et al., 2026). In parallel, pretreatment microbial composition and functional genes, including pathways related to purine and MTX metabolism, have been associated with inadequate MTX response (Artacho et al., 2021). These observations support a bidirectional model in which the microbiota may contribute to response heterogeneity while pharmacological exposure continuously remodels the ecosystem. They do not establish microbiota-mediated resistance across all DMARD classes.

## 3 Direct evidence: gut dysbiosis in D2T RA

### 3.1 Direct comparison between D2T RA and E2T RA

Studies in conventional RA cohorts have associated intestinal microbial features with clinical improvement and treatment response (Gupta et al., 2021; Koh et al., 2023). Building on this evidence, Ruiz-Limón and colleagues directly compared the gut microbiota of patients with D2T RA and E2T RA. The analysis was nested within a previous prospective RA cohort and included 13 patients with D2T RA and 26 with E2T RA. Fecal community composition was characterized using 16S rRNA gene sequencing, and functional potential was inferred using PICRUSt2 (Ruiz-Limón et al., 2025). *Bacteroidetes* and *Megasphaera* were relatively enriched in D2T RA, whereas *Proteobacteria*, *Pasteurellaceae*, *Haemophilus*, *Lachnospiraceae*, and *Coprococcus* were relatively enriched in E2T RA. Although the individual abundances of *Bacteroidetes* and *Firmicutes* did not differ significantly, the *Firmicutes*/*Bacteroidetes* ratio was lower in D2T RA (Ruiz-Limón et al., 2025). In analyses of clinical variables, higher Disease Activity Score in 28 joints using the erythrocyte sedimentation rate (DAS28-ESR) and prednisone use were associated with a greater probability of D2T RA, whereas a higher *Firmicutes/Bacteroidetes* ratio was associated with a lower probability. Functional predictions also suggested differences in pathways related to degradation, utilization, assimilation, and biosynthesis (Ruiz-Limón et al., 2025). This study currently provides some of the most direct human evidence in D2T RA, but the findings should be interpreted as exploratory associations rather than an established D2T RA-specific microbial signature.

### 3.2 Strength and major limitations of the evidence

D2T RA-specific evidence is currently too limited to support a stable microbial biomarker. In the principal direct study, only 13 patients with D2T RA were compared with 26 patients with E2T RA, and the cross-sectional, single-sample design could not establish whether ecological differences preceded treatment failure (Ruiz-Limón et al., 2025). Reverse causation is a major concern because chronic inflammation, disease duration, diet, comorbidity, and repeated exposure to DMARDs, glucocorticoids, antibiotics, proton pump inhibitors, and analgesics can all alter the microbiome (Aasmets et al., 2025; Gacesa et al., 2022; Vujkovic-Cvijin et al., 2020). Methodological resolution is another constraint: 16S rRNA sequencing characterizes taxonomic composition, while PICRUSt2 estimates rather than measures functional potential (Douglas et al., 2020; Ruiz-Limón et al., 2025). The study is therefore valuable as a disease-specific signal, but the signal remains exploratory until reproduced with longitudinal sampling, shotgun metagenomics, metabolomics, objective inflammatory phenotyping, and detailed medication data.

### 3.3 From direct observations to a testable chain of evidence

Because direct D2T RA data are sparse, the evidence chain must be explicit. The D2T RA–E2T RA comparison supplies the disease-specific signal (Ruiz-Limón et al., 2025). Conventional RA and experimental studies then provide mechanistic plausibility, including microbial antigen cross-reactivity and barrier/immune pathways (Chriswell et al., 2022), while treatment-response cohorts link baseline microbial features to initial DMARD response (Artacho et al., 2021; Danckert et al., 2024). Intervention data, such as the inulin trial in conventional RA, indicate possible translational directions but do not establish efficacy in D2T RA (Tabatabaeyan et al., 2025). We therefore treat each layer according to its directness to D2T RA rather than merging them into a single evidence tier.

## 4 From RA-associated dysbiosis to hypothesized functional microbial states in refractory disease

### 4.1 The broader landscape of dysbiosis in RA

RA-associated dysbiosis is heterogeneous across disease stages, treatment states, and populations. Signals involving *Prevotella copri* or *Prevotellaceae* are most evident in at-risk or untreated early disease and are not universal across established RA (Luo et al., 2023; Rooney et al., 2024; Scher et al., 2013). Metagenomic studies have also identified site-spanning oral–gut shifts, including reduced *Haemophilus* and increased *Lactobacillus salivarius*, with partial normalization after treatment (Zhang et al., 2015). Other cohorts report changes in *Collinsella, Eggerthella, Faecalibacterium, Lachnospiraceae*, and *Oscillospiraceae*, but the direction and magnitude vary (Chen et al., 2016; Nurgaziyev et al., 2024). Longitudinal RA studies further show that treatment can remodel community structure, short-chain fatty acid (SCFA) profiles, and host immune networks (Koh et al., 2023; Marazzato et al., 2022). These inconsistencies argue against a taxon-based definition of refractory disease. We therefore use “dysbiosis” for community-level ecological disruption and focus the proposed states on functional pathways, metabolites, host phenotypes, and treatment response rather than on individual genera (Wang et al., 2022).

### 4.2 Hypothesized inflammation-amplifying functional state

Three converging lines of evidence motivate the inflammation-amplifying state: RA cohorts show impaired barrier integrity and greater exposure to microbial products; serum lipopolysaccharide (LPS) bioactivity tracks disease activity and remission probability; and experimental arthritis demonstrates TLR4-dependent mucosal Th17 polarization (Audo et al., 2023; Parantainen et al., 2022; Rogier et al., 2017). These findings do not establish a D2T-specific phenotype, but together they support a testable hypothesis in PIRRA. The proposed state is defined by a combination of pro-inflammatory microbial function, barrier failure, persistent pathogen-associated molecular pattern sensing, and amplification of innate/Th17 responses rather than by expansion of a single organism (Huang et al., 2024; Seymour et al., 2023). Barrier disruption can increase access of LPS and other microbial products to immune-sensing compartments (Chen et al., 2016), while TLR4-linked signaling can promote IL-1β, IL-6, and IL-23 production and favor sustained Th17 activity (Rogier et al., 2017). Intestinal Th17 cells may also acquire T follicular helper-like properties, providing a plausible route from mucosal activation to extraintestinal autoimmunity (Fan et al., 2025). In D2T-PIRRA, the central prediction is not simply “more dysbiosis,” but persistent coupling of ecological disruption to barrier injury, microbial-product exposure, and imaging-confirmed synovitis despite treatment. This requires prospective measurement of all four domains in the same patients.

### 4.3 Hypothesized immune-tolerance-deficient functional state

Three lines of evidence support an immune-tolerance-deficient state: human RA data show reduced fecal butyrate/propionate and depleted SCFA-producing networks; experimental studies show that SCFAs support epithelial repair and Treg/Tfr differentiation; and the direct D2T RA comparison found relative enrichment of *Lachnospiraceae* and *Coprococcus* in E2T RA (Blank et al., 2026; Ruiz-Limón et al., 2025). The hypothesis therefore concerns loss of metabolic function, not depletion of any universally “protective” taxon. *Faecalibacterium prausnitzii* can alter SCFA production and IL-17-associated responses (Moon et al., 2023), and *Eubacterium rectale* attenuated experimental arthritis while restoring butyrate and regulatory immune-cell balance (Liu X. et al., 2025). Butyrate-related interventions can increase FOXP3^+^ Treg cells and reduce Th17-related populations, whereas epithelial injury and reduced Treg/Tfr populations are linked to inflammatory activity in experimental or early RA settings (Cao et al., 2024; Lyu et al., 2024; Wu et al., 2024). Because multiple taxa can supply the same metabolite, the relevant unit is functional redundancy and measured metabolite output. In D2T-PIRRA, the testable prediction is that persistently low SCFA output and incomplete regulatory-cell recovery accompany failure to re-establish mucosal and systemic immune tolerance after therapy.

### 4.4 Hypothesized poor-drug-response-associated functional state

Evidence for a poor-drug-response-associated state comes from three sources: pretreatment microbiome-response associations in DMARD-naive RA, *ex vivo*/experimental evidence that gut communities can modify methotrexate (MTX) exposure or efficacy, and longitudinal observations that antirheumatic and concomitant medications remodel the ecosystem (Artacho et al., 2021; Danckert et al., 2024; Nayak et al., 2021). This evidence supports a hypothesis about response heterogeneity, not a shared mechanism of resistance to multiple DMARD classes. Pretreatment community composition, functional genes, and enterotypes have been associated with MTX response (Artacho et al., 2021; Qiao et al., 2023), and broader fecal/salivary signals have been associated with subsequent DMARD response (Danckert et al., 2024). Once treatment begins, MTX can perturb conserved bacterial metabolic pathways in a strain-dependent manner, while prior and current medications can leave persistent ecological signatures (Aasmets et al., 2025; Nayak et al., 2021). Poor responders may also show differences in barrier injury and inflammatory mediators (Zaragoza-García et al., 2023). The resulting state is therefore dynamic: baseline microbial function may be linked to initial non-response, and treatment exposure may subsequently reshape the same ecosystem. At present, the strongest evidence concerns MTX; direct evidence for microbiota-mediated multi-class b/tsDMARD resistance is insufficient.

### 4.5 Relationship, evolution, and testability of the hypothesized functional states

The three states should be viewed as partially overlapping axes rather than mutually exclusive categories. A patient may enter treatment with a poor-drug-response-associated profile and, after repeated non-response, mechanism switching, or glucocorticoid exposure, develop ecological changes that reinforce barrier dysfunction and microbial-product sensing. In parallel, persistent inflammation or dietary/medication effects could reduce SCFA output and regulatory immune recovery, increasing overlap with the tolerance-deficient state. The reverse sequence is also possible: pre-existing barrier or metabolic abnormalities may precede and contribute to poor response. These trajectories are hypotheses, not established natural-history stages. Table 1 therefore frames each state around a core research question rather than a diagnostic rule. Prospective D2T RA cohorts should use serial shotgun metagenomics, metabolomics, barrier markers, peripheral and synovial immunophenotyping, and imaging-confirmed synovitis to determine whether reproducible clusters or continuous state scores emerge and whether transitions predict treatment outcomes (Holers et al., 2024; Hur et al., 2025; Li et al., 2025).

**TABLE 1:** Hypothesized functional microbial states in difficult-to-treat rheumatoid arthritis (proposed endotypes for prospective validation).

Hypothesized functional state	Defining functional features	Candidate measurements	Clinical hypothesis, validation requirement, and core research question
Inflammation-amplifying	Barrier disruption; increased microbial-product sensing; TLR/NF-κB and Th17 activation	Shotgun metagenomics; LPS bioactivity, LBP and sCD14; zonulin-related markers; Th17/Treg profiling; ultrasound or MRI synovitis	Hypothesized enrichment in PIRRA. Validate temporal association with objective inflammation and response across DMARD classes. Core question: Do baseline barrier dysfunction and microbial-product exposure predict persistent imaging-confirmed synovitis during sequential treatment?
Immune-tolerance-deficient	Reduced SCFA-producing capacity; impaired epithelial support; inadequate Treg/T follicular regulatory recovery	Targeted fecal and serum SCFAs; butyrate-pathway genes; barrier proteins; Treg/Tfr/Tfh phenotyping	Test whether functional SCFA deficiency precedes persistent inflammation and whether metabolite restoration engages the predicted immune pathway. Core question: Do low SCFA output and incomplete Treg/Tfr recovery precede persistent synovitis?
Poor-drug-response-associated	Baseline microbial functions associated with non-response plus treatment-driven ecological remodeling	Pretreatment metagenomics and metabolomics; *ex vivo* drug depletion; drug-metabolism genes; serial sampling after treatment changes	Current evidence is strongest for MTX. Validate drug-specific prediction, calibration, external validation, reproducibility, and incremental clinical utility. Core question: Do pretreatment microbial functions improve prediction of drug-specific non-response beyond clinical variables, and are they reproducible across cohorts?

## 5 Mechanistic links between gut dysbiosis and persistent inflammatory refractoriness

### 5.1 Intestinal barrier disruption and translocation of microbial products

The intestinal barrier is the critical interface between the luminal ecosystem and systemic immunity. Under physiological conditions, tight-junction proteins including occludin, claudins, and the zonula occludens family restrict the paracellular entry of microorganisms and their products into host tissues (Chelakkot et al., 2018). In RA-associated dysbiosis, reduced SCFA-producing capacity and zonulin-related changes in permeability may jointly disrupt tight-junction integrity and increase intestinal permeability (Tajik et al., 2020). Human RA studies have reported lower colonic mucosal ZO-1 expression together with elevated serum LPS-binding protein (LBP) and soluble CD14 (sCD14) (Audo et al., 2023). Once the barrier is compromised, microbe-associated molecular patterns, including LPS, bacterial DNA, and peptidoglycan, become more accessible to mucosal immune surveillance and may enter the circulation under pathological conditions (Dunyach-Remy et al., 2025; Ghosh S. S. et al., 2020; Huang et al., 2019). LBP, sCD14, and LPS bioactivity are indirect markers of microbial exposure or translocation and should not be interpreted as direct evidence that viable bacteria have crossed the intestinal barrier. Of note, LBP and sCD14 declined after treatment in bDMARD responders but not in non-responders or patients treated with csDMARDs (Audo et al., 2023). This observation suggests that persistent mucosal-derived stimulation may coexist with residual inflammation in a subset of patients with RA, although longitudinal studies are required to establish the direction of causality. In a refractory setting, persistent barrier failure would be important because it could maintain a continuous source of upstream inflammatory input even when downstream cytokine pathways are pharmacologically suppressed. A critical D2T-PIRRA test is whether persistently abnormal LBP/sCD14 or barrier markers predict ongoing ultrasound synovitis across sequential treatment changes.

### 5.2 TLR/NF-κB signaling, NOD-like receptors, and sustained innate immune activation

When gut-derived pathogen-associated molecular patterns reach immune-sensing compartments, pattern-recognition receptors can convert microbial signals into pro-inflammatory transcriptional programs. Bacterial lipoproteins induce B-cell-activating factor through TLR2/MyD88/JNK signaling and activate nuclear factor-κB (NF-κB), thereby promoting Th17-related responses directly or indirectly (Im et al., 2020). LPS can stimulate macrophages and dendritic cells to produce IL-1, IL-6, and TNF-α through the TLR4/NF-κB axis (Park et al., 2013). Cytosolic nucleotide-binding oligomerization domain-containing protein 2 (NOD2) recognizes bacterial peptidoglycan fragments (Kufer, 2025) and activates mitogen-activated protein kinase and NF-κB pathways through receptor-interacting protein kinase 2, promoting inflammatory mediators including IL-1β and IL-18 (Ahmad et al., 2025; Zhou et al., 2025). In myeloid cells, bacterial components may also trigger NLR family pyrin domain-containing 3 inflammasome assembly and IL-1β maturation (Baik et al., 2023). These pathways are general innate immune mechanisms, and some supporting evidence derives from non-RA diseases or experimental systems. Their relevance to D2T-PIRRA lies in the possibility that ongoing TLR/NOD/inflammasome activation creates a multi-cytokine inflammatory “floor” involving TNF-α, IL-6, IL-23, and IL-1β (Guo et al., 2018). Such distributed activation could exceed the suppressive capacity of any single downstream agent and offers a biologically plausible, but unproven, explanation for sequential failures of TNF-, IL-6-, or JAK-directed therapy in a subset of patients. Validation requires paired microbial, pathogen-associated molecular pattern, and synovial measurements across treatment transitions.

### 5.3 Th17/Treg imbalance and maintenance of autoimmunity

In the healthy intestine, a dynamic balance between Th17 and regulatory T (Treg) cells helps reconcile mucosal defense with immune tolerance (Jia et al., 2020). Experimental studies suggest that ecological disruption can coincide with enhanced Th17 responses and impaired Treg function, thereby shifting immunity toward inflammation and compromising mucosal homeostasis (Liu H. Y. et al., 2025; Zhao et al., 2021). Because similar immune phenotypes can arise from distinct community configurations (Arumugam et al., 2011), taxonomic changes should not be mapped directly onto immune function. At the cytokine level, IL-1β promotes Th17 polarization through IL-1 receptor/PI3K-mTOR-associated signaling and suppresses transforming growth factor-β-induced Foxp3 expression (Guo and Zhang, 2021). Th17/Treg imbalance is closely associated with inflammatory activity in RA (Yasuda et al., 2019). Experimental data further show that IL-1β can induce aberrant receptor activator of nuclear factor-κB ligand expression in Treg cells and generate an osteoclastogenic phenotype, whereas restoration of the Th17/Treg balance attenuates synovitis, bone erosion, and cartilage damage (Levescot et al., 2021; Yoo et al., 2020). Reduced absolute numbers of circulating Treg cells and an increased Th17/Treg ratio have been reported in D2T RA (Yan et al., 2025). Th17/Treg imbalance may therefore represent an adaptive immune link between disrupted mucosal tolerance and persistent autoimmunity. In refractory disease, a persistently Th17-biased or Treg-deficient mucosal state could allow adaptive immune activation to rebound after otherwise effective suppression of individual cytokines. The key D2T-PIRRA question is whether failure of regulatory-cell reconstitution, rather than the absolute baseline Th17/Treg ratio, tracks objective synovitis and repeated treatment failure.

### 5.4 SCFA deficiency and failure of metabolic-immune regulation

SCFAs are major microbial functional outputs linking barrier homeostasis to immune regulation. Butyrate, propionate, and acetate influence epithelial physiology. Butyrate, in particular, has histone deacetylase (HDAC)-inhibitory activity and can regulate the assembly and expression of tight-junction proteins (Korsten et al., 2023); insufficient SCFA availability may consequently reduce the epithelial capacity to contain microbial products (Wang R. X. et al., 2020). Acetate supplementation can also promote barrier repair and antimicrobial peptide expression (Yap et al., 2021). At the level of regulatory immunity, SCFAs influence the size and function of the colonic Treg pool (Smith et al., 2013). Butyrate and propionate promote FOXP3^+^ Treg differentiation through HDAC inhibition (Arpaia et al., 2013), and butyrate can enhance histone acetylation at T follicular regulatory cell-associated genes and promote functional T follicular regulatory differentiation (Takahashi et al., 2020). In myeloid cells, butyrate-mediated HDAC inhibition dampens pro-inflammatory macrophage responses to bacterial signals (Chang et al., 2014). Butyrate and propionate also suppress NF-κB-, STAT1-, and inducible nitric oxide synthase-associated inflammatory programs (Park et al., 2019) and can shift some macrophages toward an arginase-1-associated reparative phenotype (Ji et al., 2016). Most of these mechanisms derive from experimental studies and do not establish a uniform SCFA-deficiency syndrome in D2T RA. If confirmed in D2T-PIRRA, however, SCFA deficiency would be particularly relevant because it could simultaneously compromise epithelial containment, Treg/Tfr reconstitution, and restraint of inflammatory myeloid cells. This multi-layered loss of homeostatic control could make recovery harder to achieve with a single immune target even when that target is pharmacologically inhibited.

### 5.5 Molecular mimicry, citrullination, and autoantibody responses

Antigenic cross-reactivity is a plausible route by which mucosal microbes could reinforce RA autoimmunity. *Prevotella*-, *Parabacteroides*-, *Butyricimonas*-, and *Subdoligranulum*-derived antigens have been linked to cross-reactive T- or B-cell responses in RA or experimental systems (Pianta et al., 2017; Chriswell et al., 2022). Oral studies involving *Porphyromonas gingivalis* and *Aggregatibacter actinomycetemcomitans* provide complementary evidence for citrullination, antibody cross-recognition, and epitope spreading (Lundberg et al., 2008; Konig et al., 2016; Brewer et al., 2023). These oral findings are mechanistically informative but do not establish an intestinal origin. In this gut-focused review, the oral microbiome is therefore treated as indirect support for a broader mucosal autoimmunity mechanism rather than as evidence of a gut-specific D2T pathway.

### 5.6 From intestinal inflammation to refractory synovitis: a proposed integrative model

The evidence can be integrated into a working model centered on D2T-PIRRA (Figure 1). Human RA studies link barrier dysfunction and microbial-product exposure to disease activity, while experimental work shows that microbial sensing can activate several inflammatory branches in parallel (Audo et al., 2023; Parantainen et al., 2022; Rogier et al., 2017). In this model, spatially distributed gut-derived inputs repeatedly feed innate and adaptive pathways upstream of the synovium; a TNF, IL-6, JAK–STAT, B-cell, or T-cell-directed drug may therefore suppress one node without removing the external input that helps repopulate the network. This offers a plausible explanation for multi-drug failure in PIRRA, but it remains an inference. No longitudinal D2T RA study has yet connected microbial ecology, barrier function, circulating microbial signals, synovial pathobiology, and failure of multiple DMARD mechanisms in the same patients.

**FIGURE 1:**
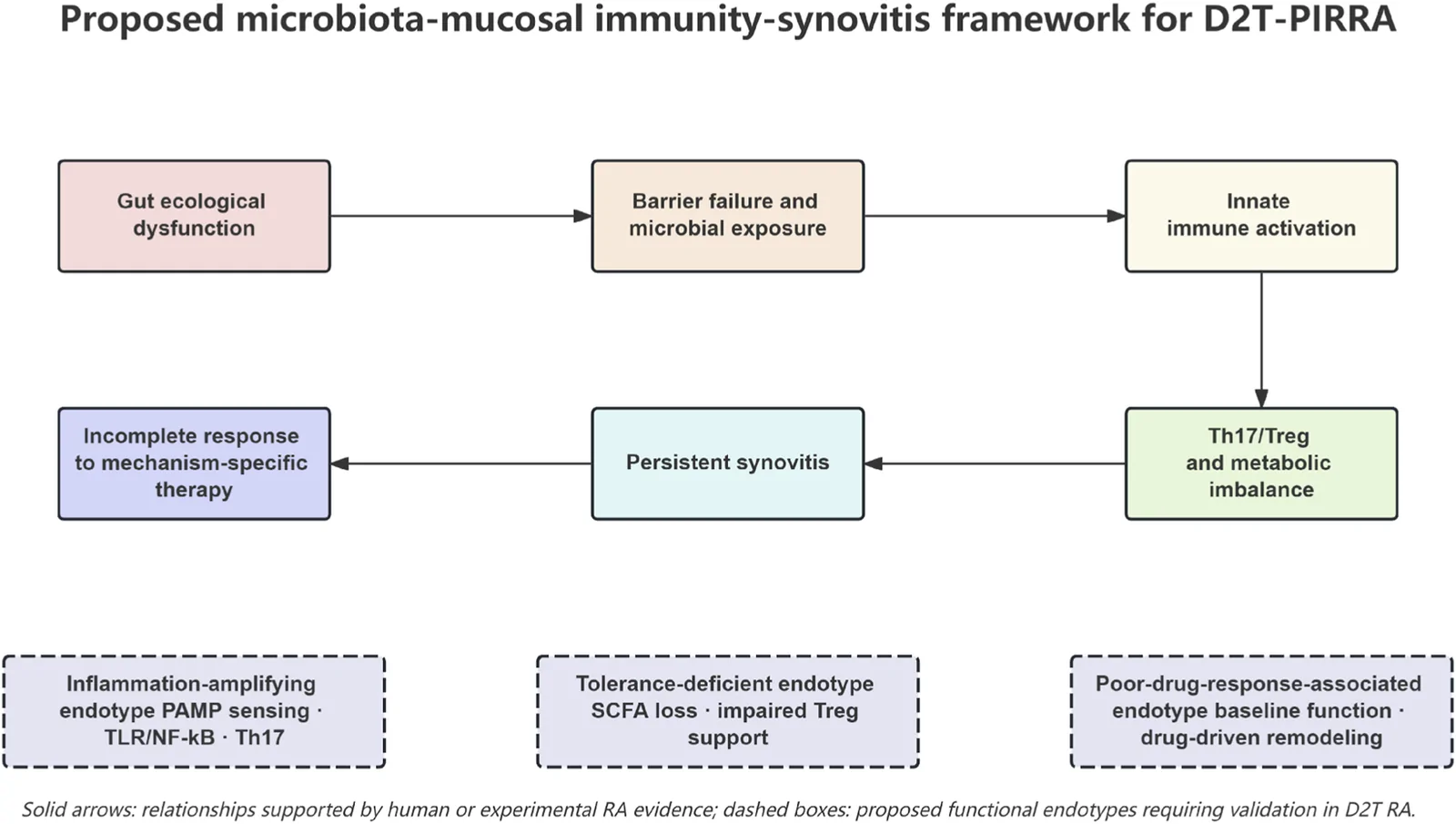
Hypothesis-generating microbiota–mucosal immunity–synovitis framework for persistent inflammatory refractory rheumatoid arthritis. Community-level gut dysbiosis may be accompanied by barrier failure, microbial-product exposure, and altered microbial metabolites, which can converge on innate and adaptive inflammatory pathways. The three proposed endotypes are hypothesized functional states, not data-derived patient classes. Solid arrows summarize relationships supported by human or experimental RA evidence; they do not imply that the complete pathway is causal in D2T RA. Dashed endotype boxes require direct prospective validation.

## 6 Gut microbiota, pharmacomicrobiomics, and treatment resistance

### 6.1 Pretreatment microbiome features and DMARD response

In newly diagnosed, DMARD-naive RA, the pretreatment microbiome may have value for therapeutic stratification. Danckert and colleagues performed shotgun metagenomic sequencing of baseline fecal and salivary samples from 144 patients and found that microbial functional pathways were more informative than individual taxonomic features for clinically important improvement at 6 weeks. Predictive performance based on salivary functional pathways was limited but statistically significant (Danckert et al., 2024). Another study associated pretreatment fecal taxonomic composition and functional potential with MTX non-response at 4 months (Artacho et al., 2021), while *Prevotella*- and *Bacteroides*-dominated baseline enterotypes were linked to differences in MTX efficacy (Qiao et al., 2023). In an RA cohort with more heterogeneous treatment exposure, baseline fecal metagenomic features were also associated with clinically important improvement over the subsequent 6–12 months (Gupta et al., 2021). These findings support the microbiome as a candidate predictive biomarker, but an association does not constitute a clinically validated prediction tool. Current models require independent external validation, calibration, and assessment of clinical utility. Approximately 10% of patients with DMARD-naive early RA may progress to refractory disease during long-term follow-up (Natalucci et al., 2025). Baseline microbial profiling could therefore add information to early D2T RA risk stratification, but its direct predictive value for a D2T RA outcome has not been demonstrated.

### 6.2 Pharmacomicrobiomics of methotrexate

MTX is the anchor csDMARD in RA, and insufficient efficacy or intolerance often prompts combination treatment and escalation to bDMARDs or tsDMARDs (Fraenkel et al., 2021; Smolen et al., 2026). Current pharmacomicrobiomic evidence suggests that intestinal microorganisms may influence luminal MTX disposition, effective exposure, immunological effects, and gastrointestinal tolerability through drug uptake, enzymatic transformation, and folate and purine metabolism (Yan et al., 2021). In human studies, Artacho and colleagues found substantial interindividual variation in *ex vivo* MTX depletion by fecal communities, and residual drug concentrations after incubation were associated with clinical improvement (Artacho et al., 2021). A cross-dataset metagenomic analysis further showed that genes related to MTX metabolism are widely distributed across gut microorganisms. Responders and non-responders did not differ substantially in the total number of relevant genes, but the organisms contributing those functions differed (Han et al., 2022). Animal experiments provide stronger causal support: microbiota depletion attenuated the anti-arthritic effect of MTX, whereas supplementation with *Bacteroides fragilis* or butyrate partially restored efficacy (Zhou et al., 2022). Experimental MTX-induced gastrointestinal injury is accompanied by community remodeling and changes in macrophage polarization, although its relevance to low-dose MTX toxicity in RA remains uncertain (Zhou et al., 2018). The reverse direction is also important. MTX directly perturbs conserved metabolic pathways in diverse human gut bacteria in a strain-dependent manner and can modify host immune activation through community remodeling (Nayak et al., 2021). Long-term exposure can alter microbial composition, metabolic function, and carboxypeptidase G2-related activity (Letertre et al., 2020). The MTX-microbiota relationship is therefore bidirectional rather than a unidirectional process in which bacteria merely metabolize the drug. Evidence from different experimental systems should be interpreted separately, and current data do not establish that the microbiota determines individual MTX response.

### 6.3 bDMARDs, tsDMARDs, and the microbiota: emerging signals and critical gaps

Compared with MTX, evidence on the pharmacomicrobiomics of bDMARDs and tsDMARDs is sparse and concentrated mainly on TNF inhibitors. In collagen-induced arthritis, etanercept reduced TNF-α and IL-6 and was accompanied by changes in community structure (Wang B. et al., 2020), but these findings cannot distinguish a direct drug effect on the microbiota from secondary changes caused by suppression of arthritis. Human studies have likewise reported etanercept-associated community changes and partial restoration of selected taxa (Picchianti-Diamanti et al., 2018), although cross-sectional designs or inadequate controls limit conclusions regarding temporal sequence and drug specificity. In another established RA cohort, TNF inhibitor users tended to have greater microbial diversity and a different community composition than users of IL-6 receptor inhibitors or abatacept, but the differences were not statistically significant; the very small number of Janus kinase inhibitor users precluded evaluation (Koh et al., 2023). A recent prospective study found that selected intestinal-barrier markers moved toward normal in patients who achieved remission after anti-TNF therapy, but community composition was not directly assessed (García-Studer et al., 2026). A 2026 systematic review across autoimmune and immune-mediated arthritis identified only 12 studies of biologic therapy and the microbiota, predominantly involving TNF or IL-17 inhibition, and emphasized the difficulty of separating direct microbial effects from changes secondary to disease control; extrapolation to RA is limited by mixed diagnoses (Rondla et al., 2026). Drug-specific prediction models for TNF inhibitors, IL-6 pathway inhibitors, abatacept, B-cell-targeted therapy, and Janus kinase inhibitors therefore remain largely absent.

Speculative interpretation: for many b/tsDMARDs, the dominant microbiome effect may be indirect, arising from suppression of systemic and synovial inflammation and restoration of barrier homeostasis rather than from direct drug–microbe chemistry. This would fit the partial normalization of barrier markers in anti-TNF responders and the lack of reproducible drug-specific microbial signatures. It may also explain why microbial changes differ between responders and non-responders within the same drug class. The evidence gap is partly structural: direct microbial drug metabolism is easier to study for orally administered MTX, whereas biologics and targeted agents require larger longitudinal cohorts to disentangle drug mechanism, disease improvement, prior therapies, glucocorticoids, diet, and baseline microbiota. Repeated sampling before and after treatment initiation, appropriate comparator drugs, and mediation analyses will be needed to test this hypothesis.

### 6.4 Microbiome confounding by glucocorticoids, NSAIDs, and concomitant medications

Interpretation of the microbiome in D2T RA is particularly vulnerable to confounding by non-DMARD medications and comorbid conditions. Patients may receive long-term or intermittent glucocorticoids, non-steroidal anti-inflammatory drugs (NSAIDs), proton pump inhibitors (PPIs), antibiotics, opioids, and other analgesic or metabolic medications because of persistent inflammation, chronic pain, gastrointestinal protection, recurrent infection, or metabolic comorbidity. Ruiz-Limón and colleagues associated prednisone use with the D2T RA phenotype (Ruiz-Limón et al., 2025), and a randomized study showed that short-term oral glucocorticoids can alter the gut microbiota and metabolic pathways (Lyu et al., 2025). NSAIDs can perturb both intestinal barrier function and the microbiota (Edogawa et al., 2018). PPI use is associated with reduced bacterial richness and enrichment of oral-derived and potentially pathogenic taxa (Imhann et al., 2016), whereas broad-spectrum antibiotics can cause substantial and partly persistent community restructuring (Palleja et al., 2018). Cross-cohort metagenomic analyses further indicate strong associations between PPIs, antibiotics, and metformin and microbial variation; opioids and oral glucocorticoids are also linked to selected taxa, and the number of medications used is itself an important variable (Vich Vila et al., 2020). Non-pharmacological factors, including diet, body mass index, age, sex, smoking, geography, disease duration, stool consistency, and recent infection, must also be recorded systematically. Otherwise, apparent disease-associated microbial features in D2T RA may merely represent the composite ecological imprint of cumulative drug exposure, comorbidities, and lifestyle. Future studies should prespecify a minimum metadata set and address confounding through restriction, matching, stratification, multivariable adjustment, and sensitivity analyses.

### 6.5 A proposed microbiota-treatment resistance feedback loop

A dynamic microbiota–treatment resistance loop can be proposed, but evidence differs across its links (Figure 2). The association between pretreatment microbial features and initial MTX/DMARD response is observational (Artacho et al., 2021). Treatment escalation, mechanism switching, and glucocorticoid exposure clearly occur after non-response, and several drugs can remodel the microbiome, establishing temporal plausibility for the next step (Aasmets et al., 2025; Ruiz-Limón et al., 2025). By contrast, the proposition that treatment-driven ecological remodeling then causes persistent barrier dysfunction, residual synovitis, and a lower probability of response to subsequent drug classes remains mechanistic inference. The decline of LBP and sCD14 in bDMARD responders is compatible with, but does not prove, this sequence (Audo et al., 2023). The closed loop has not been demonstrated. Validation requires serial sampling around each treatment transition, objective inflammatory phenotyping, and prespecified mediation or cross-lagged analyses.

**FIGURE 2:**
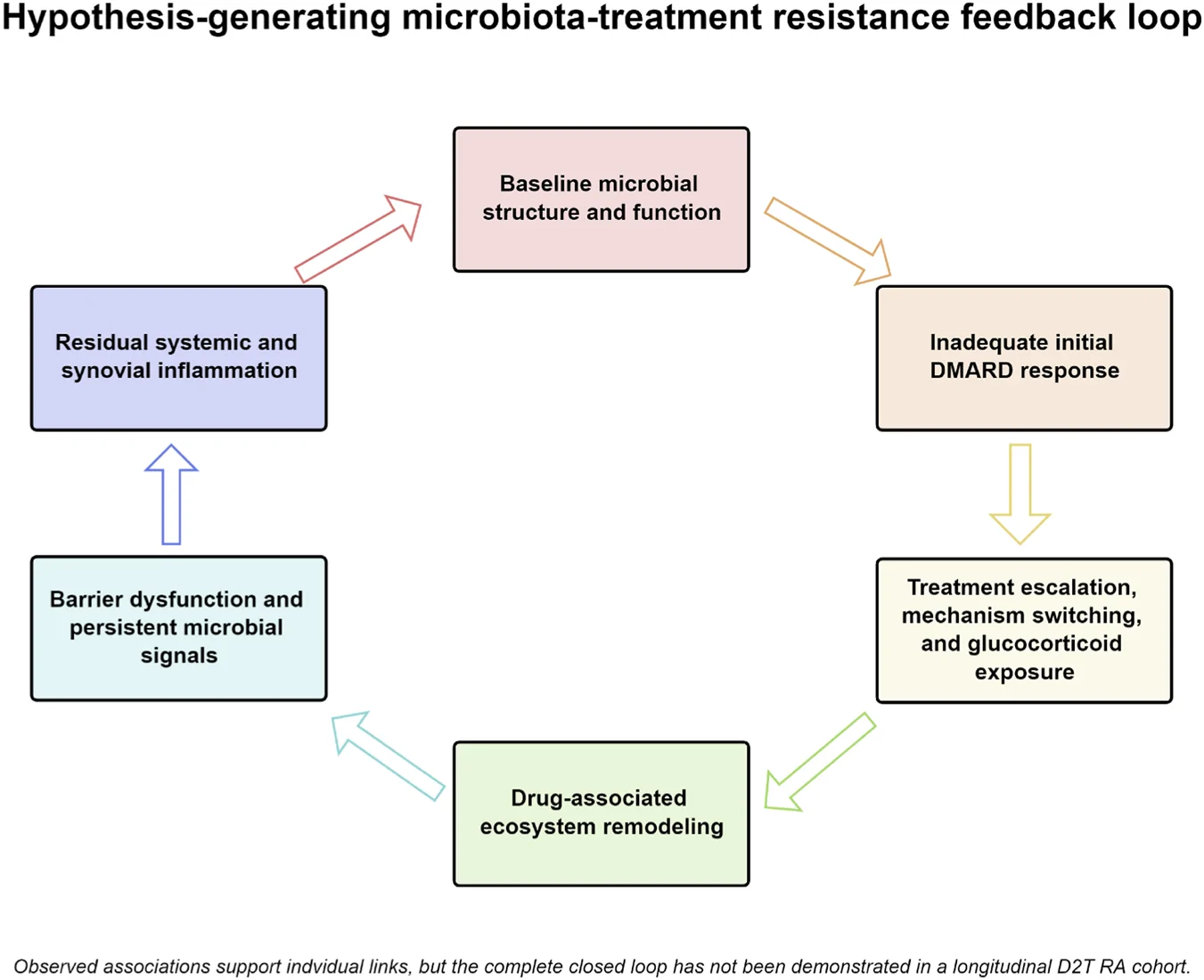
Hypothesis-generating microbiota–treatment resistance feedback loop. The figure separates evidence levels: pretreatment microbiome–response links are associative; treatment-associated ecological remodeling has temporal support; and progression from remodeling to persistent barrier dysfunction, residual inflammation, and subsequent multi-class failure remains hypothetical. Arrows indicate proposed temporal relationships and should not be interpreted as proof of causality. The complete loop has not been demonstrated in a longitudinal D2T RA cohort.

## 7 Microbiota-targeted adjunctive strategies for D2T RA

Microbiota-directed interventions are best viewed as biomarker-guided research hypotheses, not generic treatments for D2T RA. The proposed inflammation-amplifying state would most logically be studied with interventions that reduce microbial-product exposure or improve barrier function, using LPS bioactivity, LBP/sCD14, permeability markers, and Th17/Treg profiles as pharmacodynamic readouts. The tolerance-deficient state is more directly aligned with high-fiber, prebiotic, postbiotic, or metabolite strategies when low SCFA output or impaired regulatory-cell recovery is documented. The poor-drug-response-associated state requires a different design: pretreatment microbial functions should be tested as predictive or effect-modifying biomarkers rather than assumed treatment targets. These mappings are intended for trial stratification and mechanism testing only.

### 7.1 Dietary interventions and high-fiber or anti-inflammatory diets

Dietary intervention is accessible, relatively inexpensive, and compatible with long-term management, making it a potentially useful adjunct to standard pharmacotherapy (Gwinnutt et al., 2023). High-fiber diets provide fermentable substrates and may promote SCFA production. In some RA studies, these changes have been accompanied by reductions in pro-inflammatory markers and alterations in microbial composition (Dürholz et al., 2020). Randomized evidence also suggests that high-fiber multigrain supplementation may improve selected measures of disease activity, inflammation, and oxidative stress (Latif et al., 2023). The Mediterranean diet, which is rich in whole grains, legumes, fruit, vegetables, nuts, olive oil, and fish, can influence microbial composition and metabolic function through dietary fiber, polyphenols, and unsaturated fatty acids (Ghosh T. S. et al., 2020; Meslier et al., 2020). Randomized trials in RA suggest that Mediterranean-style or anti-inflammatory diets may improve selected outcomes related to disease activity, physical function, and quality of life (Raad et al., 2024; Vadell et al., 2020). Dietary effects, however, cannot be reduced to a simple assumption that more fiber is invariably beneficial. Experimental work with a specific *P. copri* strain indicates that, under particular strain and metabolic conditions, high-fiber substrates may also generate unfavorable metabolic outputs. Future precision-nutrition approaches should therefore focus on interactions among fiber type, baseline microbiota, microbial metabolites, and host phenotype rather than prescribing a uniform microbiota-directed diet for all patients with D2T RA. At present, dietary intervention may support comprehensive management but cannot replace csDMARDs, bDMARDs, or tsDMARDs. For a putative tolerance-deficient state, trial enrichment could use low fecal/serum SCFAs or reduced butyrate-pathway abundance, with restoration of these measures prespecified as target engagement.

### 7.2 Probiotics, synbiotics, and strain specificity

The effects of probiotics and synbiotics are likely to depend on specific strains, dose, formulation, and ecological context rather than on universal genus-level properties. In animal studies, *Lactobacillus casei* CCFM1074 attenuated arthritis and modulated the Th17/Treg balance; the protective effect of *Bifidobacterium adolescentis* depended on the timing of administration, and *Bifidobacterium longum* RAPO suppressed IL-17-related responses (Fan et al., 2020; Fan et al., 2021; Jeong et al., 2021). In a clinical trial, 8 weeks of *L. casei* 01 supplementation reduced DAS28 and selected inflammatory markers (Alipour et al., 2014). A separate multi-strain probiotic study found reductions in TNF-α and IL-6 without improvement in DAS28 (Cannarella et al., 2021), whereas a 12-week synbiotic intervention added to conventional therapy did not produce a clear between-group benefit in disease activity (Esmaeili et al., 2020). These findings cannot be summarized simply as evidence that probiotics are effective or ineffective. Substantial heterogeneity in strain combinations, viable-cell dose, treatment duration, background DMARD therapy, and outcome measures limits cross-study comparison (Sanchez et al., 2022). More importantly, almost all intervention trials have enrolled patients with conventional RA, whereas direct microbiome evidence in D2T RA remains primarily observational (Ruiz-Limón et al., 2025). Probiotics and synbiotics should therefore be regarded as exploratory adjuncts. Future trials should report strain-level identity, dose, viability, stability, background medications, baseline microbiota, and objective inflammatory outcomes, with particular emphasis on stratified evaluation in D2T-PIRRA. In D2T-PIRRA, probiotics or synbiotics would be most informative when linked to a predefined inflammation-amplifying or tolerance-deficient profile and accompanied by barrier, LPS/LBP, and Th17/Treg measurements.

### 7.3 Postbiotics, microbial metabolites, and bacterial extracellular vesicles

Postbiotics, microbial metabolites, and bacterial extracellular vesicles represent a pharmacological transition from altering whole communities to exploiting defined microbial effectors. According to the International Scientific Association for Probiotics and Prebiotics consensus, postbiotics are preparations of inanimate microorganisms and/or their components. Compared with probiotics that must remain viable, their composition, dose, and storage conditions may be more readily standardized (Salminen et al., 2021). Bacterial extracellular vesicles (BEVs) can be characterized by the source strain, particle size, and protein, lipid, and nucleic acid cargo, but methods for isolation, quantification, and quality control remain insufficiently harmonized (De Langhe et al., 2024). In animal models, the butyrate prodrug SerBut attenuated autoimmune arthritis, while butyrate-associated increases in 5-hydroxyindole-3-acetic acid enhanced regulatory B-cell function through aryl hydrocarbon receptor activation and suppressed arthritis (Cao et al., 2024; Rosser et al., 2020). The microbial bile-acid metabolites 3-oxo-lithocholic acid and iso-lithocholic acid can inhibit Th17 differentiation, although their therapeutic effects in RA have not been established (Paik et al., 2022). Microbial effectors are not intrinsically anti-inflammatory. Some tryptophan metabolites enhance regulatory immunity, whereas indole can promote Th17 responses, pathogenic autoantibodies, and collagen-induced arthritis (Rosser et al., 2020; Seymour et al., 2023). Outer-membrane vesicles from *Fusobacterium nucleatum* containing FadA can aggravate synovial inflammation (Hong et al., 2023). These approaches should therefore be regarded as emerging pharmacological opportunities rather than near-term clinical therapies. Development will require rigorous characterization of dose–exposure–response relationships, targeted delivery, batch consistency, toxicity, and long-term safety. State-linked development would require selecting a defined metabolite or vesicle cargo against a measured pathway abnormality rather than administering products on the basis of taxonomic dysbiosis alone.

### 7.4 Fecal microbiota transplantation and individualized microbiota intervention

FMT can remodel the recipient ecosystem at community and strain levels, but evidence in RA is restricted to a single case report and cannot separate an intervention effect from concomitant treatment or spontaneous fluctuation (Zeng et al., 2020). Across other diseases, donor-strain engraftment depends on both donor and recipient ecology and is associated with clinical success (Ianiro et al., 2022). Safety is the dominant concern in D2T RA because many patients have received multiple immunomodulators and may remain exposed to glucocorticoids. Transmission of multidrug-resistant organisms through inadequately screened material has caused bacteremia and death (DeFilipp et al., 2019), and current guidance does not support routine microbiota-based therapy in severely immunocompromised individuals (Peery et al., 2024). FMT should therefore be limited to rigorously governed clinical trials with comprehensive donor screening, antimicrobial-resistance testing, product traceability, and prospective adverse-event surveillance (Cammarota et al., 2019). Table 2 summarizes the translational readiness of microbiota-directed strategies; none currently justifies replacement or delay of evidence-based DMARD treatment. Because FMT acts broadly, it is poorly matched to the current state model unless trials prespecify the ecological defect to be corrected and serially demonstrate target engagement; no existing biomarker currently supports state-specific patient selection.

**TABLE 2:** Translational readiness and state-linked biomarker hypotheses for microbiota-directed adjunctive strategies in difficult-to-treat rheumatoid arthritis.

Approach	Current evidence in RA	Main limitations and safety issues	Position in D2T RA and state-linked biomarker hypothesis
Dietary intervention	Small trials suggest improvements in selected inflammatory, functional, or quality-of-life outcomes; microbiome mediation is not consistently demonstrated	Heterogeneous dietary patterns, adherence, background therapy, and context-dependent microbial metabolism	Supportive care only. Most plausibly aligned with a tolerance-deficient state; enrich mechanistic trials for low fecal/serum SCFAs or reduced butyrate-pathway capacity and require metabolite target engagement
Probiotics and synbiotics	Strain-specific animal effects and inconsistent clinical effects on DAS28 and cytokines	Strain, dose, viability, duration, baseline ecology, and concomitant therapy vary substantially	Exploratory adjunct. Most relevant to inflammation-amplifying or tolerance-deficient states; stratify by barrier/LPS-LBP signals and Th17/Treg imbalance, with strain-resolved analysis
Postbiotics and microbial metabolites	Preclinical evidence supports selected SCFA, tryptophan, and bile-acid pathways	Dose–exposure relationships, delivery, off-target effects, manufacturing consistency, and long-term safety remain unresolved	Promising pharmacological platform but not routine therapy. Match the defined metabolite or postbiotic to a measured pathway deficit (for example, low SCFA output) and prespecify immune/barrier target engagement
Bacterial extracellular vesicles	Mechanistic studies show both immunoregulatory and pro-inflammatory effects depending on source and cargo	Isolation, quantification, cargo definition, biodistribution, potency assays, and toxicity are not standardized	Early preclinical stage. Potentially relevant to inflammation-amplifying signaling, but source strain and cargo determine direction of effect; candidate biomarkers include microbial-product sensing and innate inflammatory readouts
Fecal microbiota transplantation	One RA case report; no controlled D2T RA efficacy evidence	Infection and antimicrobial-resistance transmission, variable engraftment, donor dependence, and heightened risk under immunosuppression	Research-only intervention. No validated state-specific indication; if studied, use rigorously phenotyped PIRRA, prespecify the ecological defect to be corrected, and demonstrate serial target engagement

## 8 Discussion and future directions

### 8.1 Central interpretation and boundaries of the evidence

Direct D2T RA microbiome evidence remains limited to small observational data, so the microbiota cannot be presented as a cause of D2T RA or multi-class drug failure (Ruiz-Limón et al., 2025). The mechanistic framework is deliberately centered on PIRRA, where objective synovitis provides a biologically coherent endpoint for testing barrier dysfunction, microbial-product sensing, immune imbalance, and altered microbial metabolites. NIRRA remains clinically important but is outside the core causal model; gut–brain effects may modify pain or fatigue, yet current evidence is insufficient to explain non-inflammatory refractoriness (Buch et al., 2021). Pretreatment microbial profiles are associated with early DMARD response, especially MTX, but have not been validated as predictors of EULAR-defined D2T RA (Artacho et al., 2021; Danckert et al., 2024). Accordingly, the proposed states and feedback loop should be read as testable organizing hypotheses rather than clinical classifications.

### 8.2 Research priority 1: rigorous definition and inflammatory stratification

Future microbiome studies in D2T RA should first standardize case ascertainment. Participants should strictly meet the EULAR definition, with complete documentation of csDMARD failure, inadequate response to bDMARDs or tsDMARDs with different mechanisms, persistent disease manifestations, and management difficulty, thereby avoiding the misclassification of patients with generally high disease activity or extensive drug exposure as D2T RA (Nagy et al., 2021; Nagy and Buch, 2025). Mechanistic stratification into PIRRA and NIRRA should then be applied (Buch et al., 2021). PIRRA should be defined primarily by objective evidence, including swollen joints, elevated CRP or erythrocyte sedimentation rate, and ultrasound- or magnetic resonance imaging-confirmed synovitis, with synovial histology incorporated where feasible. NIRRA assessment should systematically address fibromyalgia, pain sensitization, fatigue, structural damage, psychological factors, and comorbidities (David et al., 2024; Giollo et al., 2026). An optimal comparator framework would include healthy controls, untreated early RA, E2T RA, and D2T RA stratified according to inflammatory phenotype. Only such a design can separate microbial shifts shared across RA, changes attributable to treatment exposure, and signals genuinely associated with inflammatory refractoriness.

### 8.3 Research priority 2: longitudinal multi-omics rather than single-time-point comparisons

Following rigorous phenotyping, research should move from single fecal comparisons to synchronized longitudinal collection of multiple data layers from the same patients. PICRUSt2 infers microbial functional potential from 16S rRNA sequencing (Douglas et al., 2020); core cohorts should therefore prioritize shotgun metagenomics and collect fecal metabolomic data, peripheral immune-cell profiles, circulating inflammatory mediators, intestinal permeability markers, and indices of microbial exposure or translocation such as LBP and sCD14 at the same time points. Serum LBP and sCD14 reflect changes associated with exposure to bacterial products in RA (Audo et al., 2023), whereas musculoskeletal ultrasound provides an objective measure of active synovitis and supports PIRRA/NIRRA differentiation (David et al., 2024). Sampling should cover pretreatment baseline, early response assessment, treatment modification, and long-term follow-up, with parallel documentation of diet, body mass index, stool consistency, infection, antibiotics, and other medications. Only by placing microbial changes, metabolic outputs, immune states, and treatment responses on the same temporal axis can investigators determine which features precede treatment failure and which are consequences of disease or pharmacological exposure.

### 8.4 Research priority 3: prediction models and stratified intervention trials

Future research can advance both D2T RA risk prediction and stratified interventions in D2T-PIRRA. Prediction studies could define the first occurrence of EULAR-classified D2T RA within a prespecified follow-up period as the outcome (Nagy et al., 2021) and integrate microbial species, functional pathways, and metabolites with clinical variables including disease activity, serological status, comorbidities, previous treatment response, and drug exposure. High-dimensional microbiome data are particularly susceptible to overfitting. Analysis plans should therefore be preregistered, both internal and independent external validation should be performed, and discrimination, calibration, and clinical utility should be reported. Reporting and risk-of-bias assessment may follow TRIPOD + AI and PROBAST + AI, respectively (Collins et al., 2024; Moons et al., 2025). Intervention trials should preferentially enroll patients with D2T-PIRRA supported by objective inflammatory evidence and should position microbiota-directed treatment as an adjunct to standard DMARD therapy. Protocols and reports should follow SPIRIT 2025 and CONSORT 2025, respectively (Chan et al., 2025; Hopewell et al., 2025). In addition to DAS28, trials should prespecify objective outcomes such as CRP or erythrocyte sedimentation rate, ultrasonographic synovitis, glucocorticoid reduction, treatment persistence, and adverse events. Parallel microbial and metabolite measurements are required to determine whether the intervention engages the intended ecological mechanism.

### 8.5 Limitations and the need for cautious interpretation

Several limitations constrain this framework and should be considered together. D2T RA-specific microbiome evidence is based on small cohorts, with the principal direct comparison including 13 D2T and 26 E2T patients; temporal direction and causal relationships are therefore unresolved (Ruiz-Limón et al., 2025). Medication exposure, diet, geography, stool characteristics, body mass index, smoking, infection, and comorbidity can confound microbial associations (Aasmets et al., 2025; Gacesa et al., 2022; Vujkovic-Cvijin et al., 2020). The three proposed endotypes are hypothesized functional states, not clusters derived from unsupervised data, and the feedback loop has not been demonstrated within a single longitudinal cohort. Mechanistic support often comes from conventional RA, other immune-mediated diseases, or animal models, and oral-microbiome evidence is indirect for a gut-centered model. Finally, this is a targeted narrative review without a preregistered systematic protocol or formal risk-of-bias assessment, so selection bias remains possible. None of these concepts should currently be used to diagnose D2T RA, guide drug switching from a single microbial marker, or select patients for microbiota-directed therapy.

## 9 Conclusion

D2T RA is a heterogeneous clinical state in which persistent inflammation, non-inflammatory symptoms, cumulative pharmacological exposure, and management complexity can coexist. The limited direct human evidence shows ecological differences between D2T and E2T RA but does not establish a microbiome cause of multi-class treatment failure. When conventional RA cohorts, mechanistic studies, and pharmacomicrobiomic data are considered together, the gut microbiota is most plausibly viewed as an upstream modifier of mucosal and systemic inflammation, with the clearest testable relevance to PIRRA.

Accordingly, the inflammation-amplifying, immune-tolerance-deficient, and poor-drug-response-associated concepts are best treated as hypothesized functional states that may overlap and evolve over time. Their value will depend on whether longitudinal D2T RA cohorts can reproduce them using objective PIRRA/NIRRA phenotyping, shotgun metagenomics, measured metabolites, immune profiling, and complete drug-exposure data. Microbiota-targeted approaches remain investigational adjuncts; future trials should link each intervention to a prespecified state and biomarker of target engagement rather than apply them indiscriminately.

## Glossary

ACPA: anti-citrullinated protein antibody
ACR: American College of Rheumatology
BEVs: bacterial extracellular vesicles
bDMARDs: biological disease-modifying antirheumatic drugs
CRP: C-reactive protein
csDMARDs: conventional synthetic disease-modifying antirheumatic drugs
D2T RA: difficult-to-treat rheumatoid arthritis
DAS28: Disease Activity Score in 28 joints
E2T RA: easy-to-treat rheumatoid arthritis
ESR: erythrocyte sedimentation rate
EULAR: European Alliance of Associations for Rheumatology
FMT: fecal microbiota transplantation
HDAC: histone deacetylase
IL: interleukin
JAK: Janus kinase
LBP: lipopolysaccharide-binding protein
LPS: lipopolysaccharide
MRI: magnetic resonance imaging
MTX: methotrexate
NF-κB: nuclear factor kappa B
NIRRA: non-inflammatory refractory rheumatoid arthritis
NOD2: nucleotide-binding oligomerization domain-containing protein 2
NSAIDs: non-steroidal anti-inflammatory drugs
PIRRA: persistent inflammatory refractory rheumatoid arthritis
PPIs: proton pump inhibitors
RA: rheumatoid arthritis
SCFAs: short-chain fatty acids
sCD14: soluble CD14
Tfh: T follicular helper
Tfr: T follicular regulatory
TLR: Toll-like receptor
TNF: tumor necrosis factor
Treg: regulatory T cell
tsDMARDs: targeted synthetic disease-modifying antirheumatic drugs
